# Inter-Allied Scientific Food Commission

**Published:** 1918-06-29

**Authors:** 


### The Hospital

The Workers' Newspaper of Administrative Medicine and Institutional Life, Administration, National Insurance and Health.

No. 1673, Vol. LXIV. SATURDAY, JUNE 29, 1918. PRICE ONE PENNY

### INTER-ALLIED SCIENTIFIC FOOD COMMISSION.

The need for an organised co-ordination of the resources of the Allied nations fighting against the German peril is a growing quantity. Finance early became a department in which this necessity manifested itself, and the supply of munitions of war soon followed. More recently the demand for a regulated and ordered union of military forces was recognised, and, at least in some degree, diplomacy has felt a similar pressure. The prolongation of the struggle, and the menace of the submarine, have forced the conviction that the same principle must now be applied to food supplies, and it is out of this conviction there issued the decision, taken at an Inter-Allied Conference held at Paris in November 1917, to set up an International Scientific Food Commission. The Commission includes representatives of the United Kingdom (Professor E. H. Starling and Professor T. B. Wood), the United States (Professor Chittenden and Professor Lush), France (Professor Grey and Professor Langlois), Italy (Professor Botazzi and Professor Pagliani), and Belgium (Professor Hulot). At the first meeting, held at Paris in March 1918, the Commission determined the procedure to be adopted for the collection of the necessary information, and fixed a standard of the minimum food requirements of "the average man." It was agreed that a man of average weight (seventy kilos, or 154 pounds) doing average work during eight hours a day requires daily food having an energy value of 3,300 calories; and, further, that if the supply of this amount of food became impossible a reduction of 10 per cent. could be supported for some time without injury to health. The proportionate allowance to be assigned to women, and to children of different ages, was also decided. At a second meeting, held in Rome in April last, the whole question of the food supplies and requirements of the Allies was discussed, and decisions governing the general situation were taken. These decisions are the necessary preliminary to a survey of the position by which it will be possible to determine, in reference to each country, either, on the one hand, the amount of food which must be imported in order to secure the supplies needed by the population, or, on the other, the exportable surplus which the country can spare.

A finding of great interest is the decision of the Commission not to fix a minimum meat ration, seeing that no absolute physiological need exists for meat, since meat proteins can be replaced by other proteins, either vegetable in origin or those contained in milk, cheese, and eggs. To some readers such a conclusion may have a startling quality; but it must be remembered that the Commission is not charged with the duty of defining the most desirable food scheme for mankind, but rather with a scheme which will permit life and activities to continue without definite and measurable prejudice, and which, moreover, has to be arranged at a time of difficult and limited supply. It is not the optimum, but the safe minimum, which is the governing factor. But while meat may be left in this position, it is quite otherwise in regard to fats. For these there is no substitute. Hence the Commission has resolved to fix as a "desirable minimum" seventy-five grams, that is about 2⅝ ounces, of fat per average man per day. Whether "desirable minimum" is quite a happy term may be doubted; but we may suppose that it indicates what is "desirable" in existing circumstances—not what is exactly to be desired in the best interests of the individual, but a point below which the supply ought not to be allowed to fall, in view of the conditions of the time. The fats may be obtained partly from vegetable source and partly from animal sources, and the suggestion is made that to secure the figure indicated it may be necessary to maintain a certain stock of animals to furnish the required fat.

As a basis for deciding the supplies needed by each country the Commission has determined a standard of "man value," and has estimated the population of each country in terms of "average men." Obviously the figures thus defined will announce the exact amount of food which must be provided for the adequate nourishment of the total population of each of the countries to which the calculation has been applied. In reference to cereals and other products of the soil, a calculation has been made of the amount of home production in each instance, and this has to be associated with the needs of men on the one hand and of animals on the other. It is advised that each delegation, in calculating the amount of calories available for men, should assign to men the maximum possible proportion of all cereals except oats. Another resolution is that a uniform average milling extraction of 85 per cent. for wheat should be adopted throughout the Allied countries, allowing a variation from 80 per cent. in summer to 90 per cent. in winter, and recognising that the standard can only apply to the United States as regards their internal consumption, and then only in time of scarcity.

The Commission recognises that while the various countries must reserve the maximum possible of the supply of cereals for the use of man, the methods adopted to secure this end will probably vary. They advise that in the fixing of prices it is the prices of animal products which should be limited, rather than those of such vegetable productions of the soil as may serve equally well for feeding men and animals. The Commission emphasises the need for the application of economic pressure with a view to influence the producers of food in the selection of the fashion in which they will utilise their opportunities. Finally the Commission urges that propaganda having for its object the encouragement of food production and of economy in the use of food should be organised and directed by men of science well acquainted with the subject. With this recommendation we cordially agree. But we will venture to add the suggestion that steps should be taken to secure that the advice of such experts shall be translated into terms that may be understood by the common people. When, as now, supplies are limited and efficient utilisation of them is a necessity of national existence, the direction of the expert is essential. His task is to tell us not what is "desirable," but what is possible in unusual and critical circumstances. But if he wishes to be effective he must come down from his pedestal, and must utter his advice and direction in such terms that he who runs may read. His elaborate reports may be necessary for the Government Departments, but the mind of the "average man" will put them on one side with a yawn.

